# Sodium-glucose co-transporter-2 inhibitors in the intensive care unit setting: Do we really need sodium increase, especially in sepsis?

**DOI:** 10.1186/s13054-023-04501-x

**Published:** 2023-05-26

**Authors:** Dimitrios Patoulias

**Affiliations:** Second Department of Internal Medicine, European Interbalkan Medical Center, Thessaloniki, Greece

Dear Editor,

Mårtensson and colleagues recently published the results of their very interesting pilot, observational study regarding the safety and efficacy of empagliflozin, a sodium-glucose co-transporter-2 (SGLT-2) inhibitor, in subjects with underlying type 2 diabetes mellitus admitted to the Intensive Care Unit (ICU) [1]. Researchers demonstrated that subjects administered empagliflozin, compared to control group, experienced a significant increase in sodium levels (9 vs. 3 mmol/L, *p* = 0.04), while, a marginally non-significant increase in chloride levels with empagliflozin was also shown (8 vs. 3 mmol/L, *p* = 0.052) [1]. Of note, across a wide range of prespecified, assessed outcomes, including biochemical, renal, infectious outcomes and in-hospital death, no significant difference between the two treatment groups was shown [1]. A closer look at participants’ baseline characteristics reveals that a significantly greater proportion of subjects administered empagliflozin compared to the control group had sepsis as admission diagnosis (56% vs. 6%, *p* < 0.001) [1]. An additional observation is that, as depicted in the corresponding Fig. 1, sodium levels increased in the empagliflozin from eunatremic levels (between 135 and 140 mmol/L) prior to initiation to hypernatremic levels (> 145 mmol/L) 144 h after treatment initiation [1].

The question that inevitably arises is whether we actually need such an increase in sodium levels in patients hospitalized in ICU. According to a formerly published observational study utilizing data from 207,702 ICU patients, ICU-acquired hypernatremia (defined as serum sodium > 149 mEq/L) was shown to be an independent predictor of in-hospital mortality, increasing the corresponding risk by 40%, and increased length of hospital stay [2]. Especially in patients with sepsis, hypernatremia has been shown to be associated with significantly increased mortality rates, both at the Emergency Department [3] and the ICU setting [4]. At some extent, the latter might be associated with the fact that hypernatremia has been linked with persistent inflammation, immunosuppression and catabolism, as shown in another observational study performed in the ICU setting [5].

Thus, it seems that a subgroup analysis concerning blood electrolyte levels according to baseline presence of sepsis would add significant value to the results presented by Mårtensson and colleagues, despite the low relative frequency of sepsis as prior to admission diagnosis in the control group, as acknowledged by the authors in their limitations [1]. It has to be admitted that the study performed by Mårtensson and colleagues [1] is a pilot, exploratory study, and future studies, ideally randomized controlled trials, will shed further light on the role of SGLT-2 inhibitors in the ICU setting. However, lessons learned from such exploratory studies should not be forgotten, but further clarified in future, larger studies.

## Data Availability

Not applicable.
